# Recognising the dynamic form of fire

**DOI:** 10.1038/s41598-021-89453-4

**Published:** 2021-05-19

**Authors:** Fintan Nagle, Alan Johnston

**Affiliations:** 1 CoMPLEX, University College London, London, WC1E 6BT UK; 2 School of Psychology, University of Nottingham, Nottingham, NG7 2RD UK; 3 Imperial College, Exhibition Road, London, SW7 2AZ UK

**Keywords:** Visual system, Object vision

## Abstract

Encoding and recognising complex natural sequences provides a challenge for human vision. We found that observers could recognise a previously presented segment of a video of a hearth fire when embedded in a longer sequence. Recognition performance declined when the test video was spatially inverted, but not when it was hue reversed or temporally reversed. Sampled motion degraded forwards/reversed playback discrimination, indicating observers were sensitive to the asymmetric pattern of motion of flames. For brief targets, performance increased with target length. More generally, performance depended on the relative lengths of the target and embedding sequence. Increased errors with embedded sequence length were driven by positive responses to non-target sequences (false alarms) rather than omissions. Taken together these observations favour interpreting performance in terms of an incremental decision-making model based on a sequential statistical analysis in which evidence accrues for one of two alternatives. We also suggest that prediction could provide a means of providing and evaluating evidence in a sequential analysis model.

## Introduction

Object recognition in human observers is often characterised as a process of classification on the basis of a hierarchy of image features starting with a generic set of local image measures^1^. Recent machine learning methods take a similar approach and have proved to be successful in object classification in large data sets^2^. These methods typically use supervised learning to derive a mapping between images or image features and a set of labels which describe the content of the images^3^. A recognised problem with these methods is that image properties of the training set that correlate with the category labels can be learned with the desired image properties of the target, and give rise to classification errors^4^. These errors are often very unlike those a human would make and sometimes are caused by image manipulations that are imperceptible to human observers^5^.

Humans can recognise images they have seen before in a large corpus of remembered images. There has been some debate about the number of items and detail of the information stored. If asked which of two scenes one has seen before, a decision might be arrived at on the basis of a feeling of familiarity with respect to the general nature or gist of the scene^6^. Brady et al.^7^ compared recognition memory for pairs of isolated items that differed in terms of object type (e.g. cat vs house), object exemplar (e.g. fedora vs panama hat) or object state (e.g. cup full vs cup empty). Although, the amount of detail required to distinguish the items increases from object type to object state, a large number of items were remembered in each case^7^. The fact that we can describe these items with language, avoiding the need to show them, indicates that recognition tasks can be supported by semantic or symbolic representations. This raises the question of to what extent and how we can recognise state changes in objects which confound labelling or linguistic description.

Image sequences provide additional information about objects and object properties through dynamic change and motion. To date, the study of object state recognition of moving exemplars has focussed on the biological motion of faces^8,9^ and bodies^10^, objects which are readily parameterisable as static forms^11–14^ and whose structural change can be characterised by a time series of parameter values^15^. In both cases, object motion is highly constrained, often meaningful, and admits to coding in a reduced form. In addition, each frame of an expression or gesture can contain diagnostic information. A smile can be distinguished from a frown in a single image. It is therefore important, in studying the perception and representation of dynamic information, to avoid stimuli which allow discrimination on the basis of static structure and semantic categories.

Objects and surfaces also vary in terms of their material properties such as the viscosity of liquid flow^16^, the transparency of a fluid layer^17^ or the stiffness of cloth^18^, which can be evoked well from moving dot patterns^19^, indicating a role for the pattern of motion in delivering these percepts. Work on dynamic material properties tends to focus on how the statistical properties of image change or motion fields provide an impression of some physical quality of the material. Differences in these statistical properties are thought to mediate differences in perceptual qualities. In the auditory domain, differences in sound textures, such as might be generated by a waterfall or a flock of geese, can be distinguished^20,21^. Sound textures like dynamic visual textures^22^ comprise temporally extended stationary signals. Classification is therefore likely to be based on differences in the global statistical properties of these patterns^21^. This raises the question of how we encode dynamic exemplars: individual natural dynamic patterns which differ but are generated by the same physical process and which therefore are not distinguished in terms of their global statistical properties. These patterns are common; we can think of raindrops in a puddle, waves in a harbour, the effect of a breeze on a wheat field or bees in a swarm, but they provide a challenge for understanding visual recognition.

Distinguishing two stochastically stationary dynamic patterns does not require the encoding of a sequence over that needed to capture the statistical property in question, since the statistics do not change over time. Indeed, the internal representation of the target and the stimulus input do not need to be temporally aligned, in any sense, in order to make a judgement of their similarity. Convolutional neural networks address the problem of generalisation over space by essentially duplicating the same mechanism at all locations. For spatial pattern, convolution implements a parallel process serially. However, convolution is essentially intrinsic to pattern matching in the time domain, as the visual stimulus typically updates in time. A biological visual system has to accommodate the fact that although spatially all the relevant information for a visual discrimination is present at any time point, and can be scrutinised at length, temporally, information is only present at the sensory surface for an instant. We know from work on iconic memory^23^ that visual information can be stored for a short time, but we also know from backward masking^24^ that visual information is highly degraded by subsequent stimuli even though some information may still be accessible after masking^25^. This makes it difficult to generalise models of object or spatial pattern recognition, where all relevant information is present concurrently, to temporal patterns. In addition, we need to consider real time constraints on human visual processing in developing models of dynamic visual recognition.

Given these considerations, we addressed the problem of how the visual system recognises dynamic events by asking observers to distinguish between two different natural sequences that are not easy to characterise in reduced form and that have very similar spatio-temporal statistics. We use a video of a hearth fire as the generator sequence from which we draw our events. This natural stimulus has a degree of self-similarity over time without being temporally stationary, as would be the case for a dynamic visual texture. It therefore contains separable and identifiable dynamic events. Detecting the presence of fire is an area of practical interest in computer vision where the dynamic behaviour of flame and smoke can help in distinguishing fire from image regions with a similar colour and brightness^26,27^. Flame is a very familiar dynamic object; consequently, observers are not faced with the task of encoding an entirely novel dynamic pattern.

**Figure 1:**
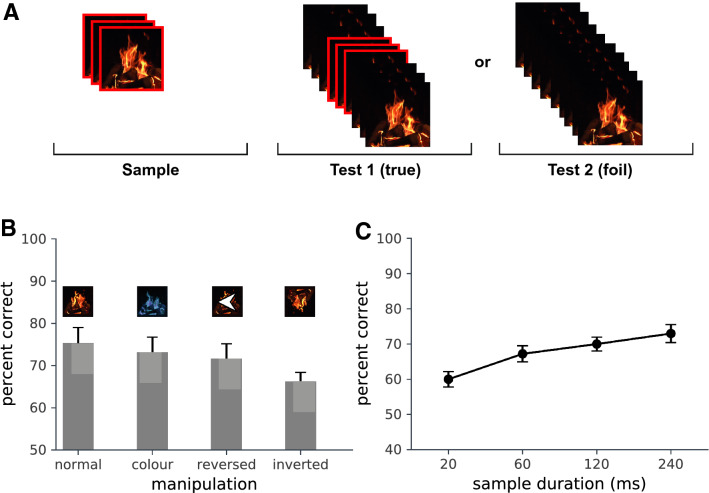
(**A**) The basic task: a short movie clip of a hearth fire (200 ms) was presented on each trial, followed by two longer sequences (300 ms), one which contained the target clip. (**B**) Experiment 1: target recognition under four manipulations. The target clip was left unaltered, hue negated, temporally reversed or spatially inverted. (**C**) Experiment 2: target detection as a function of sample length.

## Results

We first addressed the question of whether the physical characteristics of the temporal sequence influenced recognition performance. In Experiment 1 we used the 2-alternative forced choice (2AFC) paradigm: each trial contained a sample video clip followed by true and foil tests. Observers viewed a 200 ms target clip which had either been left unaltered, colour reversed in hue, spatially inverted, or played backwards. This was followed by two continuous 300 ms test clips, one of which contained the target (Fig. 1A). Observers were asked to identify from which test clip the target clip had been taken. The test clips were slightly longer (300 ms) than the embedded target (200 ms) to avoid observers basing decisions on the first or last frames. The results are shown in Fig. 1B. In each case, recognition performance was better than chance. ANOVA indicated a difference between the means ($F(3,27)=5.7$; $p<0.005$). However, performance in the hue reversal ($t=1.47$; n.s.) and reverse playback ($t=1.85$; n.s) conditions did not differ from the unaltered condition, indicating that recognition did not depend upon a pictorial match incorporating the colour of the flames and was not influenced by the direction of temporal flow. Performance was, however, reduced by spatial inversion ($t=3.55; p<0.01$), indicating that perceiving the upright form of the fire is important for recognition.

The lack of an effect of the direction of temporal flow might indicate that detection is based on encoding snapshots rather than a temporal sequence. To test this, in Experiment 2 we varied the number of frames in the target. In Experiment 1, the 200 ms target contained 10 frames. Experiment 2 was similar in design, but in this case we varied the number of target frames from 1 to 12 (1, 3, 6, 12 frames), and embedded them in test sequences of 15, 20 or 40 frames. Recognition performance (Fig. 1C) increased with sample length ($F(3,33)= 27.9$; $p<0.0005$) with a highly significant linear trend ($F(1,11)=71.0$; $p<0.0001$). Performance was unaffected by test length ($F(3,33)=0.436$; n.s.) and there was no interaction ($F(6,66)=1.06$, n.s.); thus, the data are collapsed over test length in Fig. 1C. Observers were able to detect even single-frame targets better than chance ($t= 4.39$; $p<0.005$). However, the improvement in performance with numbers of target frames suggests that recognition is not based on single snapshots alone. Motion is available from just two frames. If performance was just determined by the presence or absence of motion, we would expect an improvement from 1 to 3 frames followed by a plateau, but we would not expect a clear linear increase in performance with the number of frames, as observed. The benefit may be due to the presence of temporal information, but it may also be due to disambiguation with additional samples (more information), or the increased likelihood of the presence of a salient snapshot or dynamic feature in the target.

To determine whether dynamic events might just be encoded as a bag or sequence of snapshots, we tested in Experiment 3 whether the direction of temporal travel (video clips played forwards or backwards) could be distinguished without clear motion cues. We use temporal block sampling of the motion sequence to disrupt motion while retaining individual frames. It is possible that observers showed no effect of reversed playback on recognition in Experiment 1 because they cannot encode the temporal sequence of motion in a semi-stochastic natural stimulus like the flow of flame in fire. Alternatively, it may be that the dynamic features in flame are approximately temporally symmetric and are therefore not significantly altered by reversed playback. For instance, a flare may form, then die back, in a similar manner. In order to investigate this further, we asked observers to judge the direction of playback of the sequence. If when viewing the hearth fire observers encode a set of time stamped or ordered snapshots, then one might expect that performance in judging temporal order would improve with temporal separation, since the images will be better segregated. However, if observers utilise temporal continuity or motion, rather than simply collecting a set of ordered snapshots or key frames, then performance should be undermined by increasing temporal separation.

**Figure 2:**
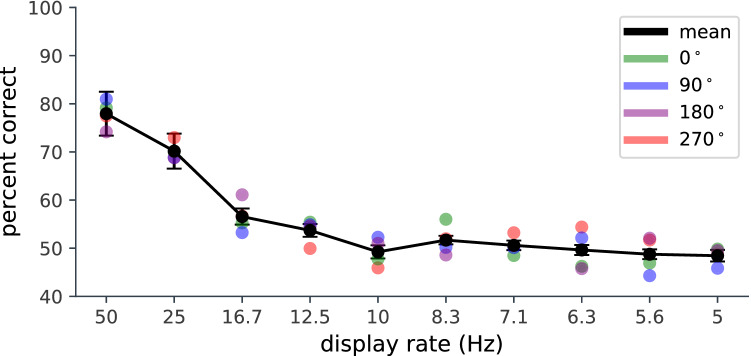
Experiment 3: The effect of display rate on playback direction discrimination. Observers indicated whether each clip presented was played forwards or reversed. Display rate describes the rate at which the frames were updated. The clips were presented at the 4 cardinal orientations. The black line is the mean of the 4 orientations.

In Experiment 3, we investigated whether local, detailed motion information was required to detect whether the fire was played normally or time reversed. We asked observers to judge whether two-second clips of hearth fire video were played forwards or backwards, while systematically degrading the motion signal by temporal block sampling. We varied display rate by updating the frame after a fixed interval, with the previous frame remaining until the next frame was presented. The experiment was repeated at 4 spatial orientations (0, 90, 180 and 270 degrees of rotation). There was an effect of display rate ($F(9,117)= 26.78$; $p<0.0005$) but no effect of orientation ($F(3, 39)= 0.1$; n.s.) so we averaged over presentation orientation. We found that performance dropped rapidly with inter-frame interval (Fig. 2) and was at chance by 100 ms (10 Hz). Errors tended to reflect an observer bias towards reporting forwards motion. This indicates that with a continuous sequence, observers can consistently distinguish between natural and reversed playback. This discrimination is not a matter of comparing the order of temporally well-separated snapshots. That observers can distinguish between forward and reverse playback at fast display rates indicates that they can encode something more than just a collection of snapshots. They also must have some naïve physical understanding of how flame behaves that survives spatial inversion, pointing to some temporal or motion property. Reversing playback does not change the low-level image statistics or the motion field speed distribution statistics. There is a global change in sign resulting from the reversal of direction of motion, but any motion frame will contain multiple directions of motion, so using motion direction as a cue would not deliver a clear indication of the direction of playback (see Supplementary Movie 2). The implication of this is that observers are sensitive to some temporally asymmetric properties of the dynamic structure. On close frame-by-frame examination, one can see that flares tend not to be temporally symmetric; rather, they tend to grow upwards over time and then extinguish transiently. This temporal characteristic would still be apparent after spatial inversion. It is clear from Experiment 3 that the temporal change in the pattern of motion and or the temporal change in form is encoded and used to distinguish forward and reversed playback. A time-stamped snapshot encoding of the pattern or motion field might allow this task to be accomplished at low display rates, but there is no evidence for that type of sequence encoding in this task.

**Figure 3:**
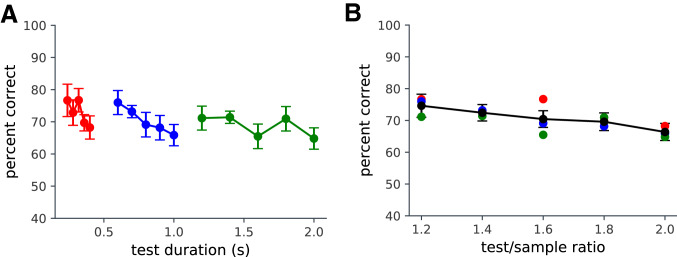
Experiment 4: the effect of test length on dynamic flame detection. (**A**) Target detection as a function of test duration for three test sample durations (red 0.2 s; blue 0.5 s; green 1 s). (**B**) Accuracy plotted as a function of test/sample ratio.

We next asked whether dynamic properties are remembered, and if so, what form does this information take? We first considered an idealised dynamic template convolution model in which the stored representation is essentially a copy of the target and the stored representation and target are relatively noise-free. On this model, an extended target movie sequence considered as a unit, slides along the input sequence with recognition derived from the degree of spatiotemporal correlation at the optimal temporal alignment. A prediction of this idealised pattern matching model is that the length of the test sequence (analogous to set size in spatial visual search^28,29^) should not affect recognition performance as there should only be one point in the sequence that provides the best match. Also, the longer the duration of the searched-for target, the less likely the occurrence of a false match.

To test this, we returned to our basic matching task, manipulating sample and test lengths in a 2AFC task. In Experiment 4, we manipulated the lengths of both the sample and test sequences. The target clip could either be 200 ms, 500 ms or 1000 ms. The target clips were embedded in test clips that were 1.2, 1.4, 1.6, 1.8 or 2 times longer than the target and the target could appear at any point in the test sequence. Significantly, when expressed in terms of the test clip length, performance declines with different rates for increasing test durations (Fig. 3). However, the rate of decline is constant when the length of the test clip is expressed relative to length of the target sequence ($F(4,44)=6.5$, $p<0.0005$). This result allows us to reject the running dynamic template or spatiotemporal cross-correlation model.

In Experiment 2, performance increased with target duration, but only a few frames were introduced before and after the target and thus there was little opportunity for false alarms. In Experiment 4, we found that recognition performance depended upon the relative length of the target and the test sequence. This is difficult to accommodate with a simple pattern matching approach. The alternative is to see identifying whether a target has been presented before or not as a process of decision making incorporating sequential analysis of evidence for and against the presence of the target^30–32^. We can explain the effect of relative length if we assume that observers accrue evidence for and against the presence of the target over the presentation interval. We can model this with a decision variable that computes a cumulative log-likelihood ratio of whether the evidence at any time point supports the presence of a target^30^. If we assume that the major determinant of a piece of evidence supporting target present is whether at that time point the sequence contains a target rather than a distractor, then increasing the target duration and test duration in proportion should not affect the log-likelihood ratio. This is consistent with the idea that a test sequence is processed on-line in a temporally sampled way and evidence is gathered and integrated until a decision boundary is reached. The overall reduction in performance as the proportion of non-target epochs in the test pattern increases is consistent with temporal uncertainty causing misses of target epochs in target present segments and the opportunity of accruing evidence of target presence (false evidence of a target) in the non-target segments.

**Figure 4:**
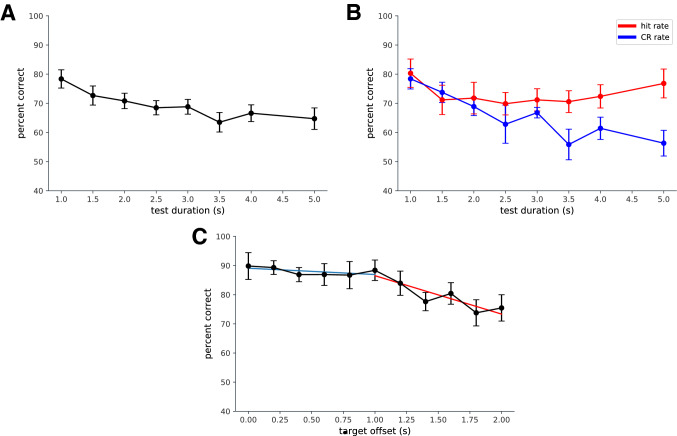
(**A**) Experiment 5: the effects of distractors and target position in a yes-no task (only one test sequence was presented on each trial). Detection accuracy is shown for a 1 s target within test clips of up to 5 s in length. (**B**) The same data as in (**A**), plotted separately for hits and correct rejections. The reduction in performance with test length is mainly due to a drop in correct rejections, rather than a drop in hit rate. (**C**) Experiment 6: we varied the position of a 1 s target within a 3 s test sequence. Later targets are detected less well.

To examine this further, we investigated the effect of search space size in more detail. In Experiment 5 we kept the target length constant (1 s) and varied the length of the test sequence. We adopted a yes-no delayed match-to-sample paradigm to obtain a measure of hits and correct rejections. The target could occur at a range of time points from the beginning to the end of the sequence. It could be preceded by between 0 and 2 s of video and followed by between 0 and 2 s of video. The foils were matched in length. Both target-present and target-absent sequences were continuous segments of video, taken from around the same time points in the full hearth fire video. We found again (Fig. 4A) that performance declined with test sequence length ($F(7,70)= 3.96$; $p<0.001$). Figure 4B shows the correct responses partitioned into hits and correct rejections. When the target was present the hit rate is relatively constant with duration with a slight rise for early and late targets. However, when the target is absent the correct rejection rate declines (i.e. false alarms increase) as the duration of the test sequence increases.

This indicates that observers made errors of inclusion rather than of omission (misses). They were willing to report the target was present in the foil and this tendency increased with test duration, which would be expected if the errors relate to the on-line decision process rather than errors at encoding. This result confirms that elements from the non-target trials appear similar enough to elements from the target sequence to induce false alarms and that the longer the test duration, the more opportunity to accumulate false evidence of the presence of a target. This is consistent with the log-likelihood ratio decision model since information supporting the evidence of the presence of the target can only lead to a false alarm in signal-absent trials^30^.

In Experiment 5, test clips were of different lengths. To investigate the time course of recognition performance more directly, in Experiment 6 we kept the target length constant at 1 s and embedded it at a range of locations in a 3 s sequence (Experiment 6). We again used a yes-no detection task. Performance depended upon delay ($F(10,20)= 4.67, p<0.001$). Splitting the function by observation, we found that performance remained constant ($F(5,30) = 0.215$, n.s.; 0.1 s) for delays up to 1 s then declined linearly (mean performance, $F(5,30)= 3.56, p<0.05$; linear trend, $F(1,6)=20.68, p<0.005$; 1.2 s) after that point (Fig. 4C). The timing of video segments giving rise to false alarms cannot be determined in this case. The decline in recognition performance indicates that either the encoding of the target becomes less precise with time or that there may be a shift in the decision criterion, characterised in accumulation models as a collapsing boundary or an urgency signal^32^. In either case, the finding is consistent with an on-line self-terminating decision-making strategy rather than a judgement made at the end of a trial.

## Discussion

McDermott et al.^21^ investigate the recognition of sound textures using a similar paradigm to that described here. They found that for sound textures with different long-term statistics, target identification (in their case, detecting the odd one out of three sequentially presented sound textures), improved with stimulus duration. However, when the textures had the same long-term statistics and were being generated by the same stochastic process, then identification performance declined with increased duration. The range of durations was comparable to that used here. The authors attribute the drop in performance to a loss of detail and reliance on global statistics for the similar textures at longer durations. In our case, the flame sequence stimuli were not temporally stationary textures, however we were also able to identify a pattern of reduced discrimination performance with increased sample duration and an increase in false positives.

We considered a number of dynamic pattern-matching strategies that participants might use to recognise a clip drawn from the hearth fire video. The first consideration is whether a decision is made subsequent to presentation of the full test pattern or patterns, or whether the search terminates with the discovery of a match. In Experiment 6, in which the target was presented at various time points within the test sequence, we found that performance declined as the time of presentation of the target in the test increased. The fact that early targets were detected more effectively suggests an on-line mode of processing in which a decision was made when some criterion for detection was reached. If the decision was made later one would expect early targets would be harder to detect due to decay of the memory trace of the early segments of the test or interference. The second possibility was that the target was stored as a dynamic sequence and the complete temporal pattern was matched to the immediate past of the test sequence at each time step. This pattern matching strategy was rejected as it predicts that target detection would be independent of test length.

The key finding that detection performance was proportional to the ratio of test length to sample length and the performance declined with a proportionate increase in the test length was explained with respect to a sequential analysis decision model in which evidence for and against the decision of target presence was evaluated over time until a decision boundary was reached. At each epoch a process would be needed to assess the sensory evidence for or against the present of a target. One might segment the target dynamic pattern into smaller dynamic templates, micro-clips, or even snapshots, which are then compared with the test in parallel. Performance in the recognition task is driven by the opportunity for false matches. The opportunity for false matches increases as the duration of the segments in the stored representation decreases, since snippets would only need to approximately match as compared to the unlikely possibility of finding a long sequence match to a signal that was not present in the test. However, there are two problems in on-line pattern matching of dynamic samples. The first is that the more finely the prepresentation is broken down into micro-clips or snapshots at encoding, the more comparisons are needed at each time step. Since both stored and test representations need to be broken down and compared, we risk a $n^2$ combinatorial explosion as the segment size decreases. The second problem is that as the segment length increases, the likelihood of a mismatch between the start and end points of the encoded segments and the test segments increases. Encoding all possible segmentations would also be computationally complex. It is possible that this strategy could be optimised, but this is not straightforward.

An interesting alternative is that rather than matching representations, we could take a predictive approach more suited to on-line processing, somewhat aligned to the concept of Granger causality^33^. This method aims to determine statistically whether one time series is useful in predicting another. We can leverage this method to support a decision on whether a segment of a dynamic sequence is known or not. According to this model, recognition is metacognitive; rather than being based on matching, it is based on evaluating, on-line, how well our target helps us predict the upcoming visual material. As the test clip is viewed, it is being predicted from both the already-viewed part of the test clip and stored knowledge of the target sequence. Evidence that the target is present accrues when the upcoming spatiotemporal pattern is better predicted by the target sequence plus the recent history of the test pattern than by the recent history of the test pattern alone. Evidence will only accrue when the searched-for pattern matches the recent history of the test sequence to a satisfactory level. One way this could proceed is by encoding a salient trigger feature and its following sequence while viewing the target and then initiating a temporally predictive process when that trigger feature or a similar feature is identified in the test pattern. This strategy differs from direct pattern matching in that rather than just provided a measure of a degree of match or correlation it provides an observable test, in terms of predicting the upcoming image sequence, of the degree to which the observer is correct in thinking that they have seen the dynamic pattern before. This should allow for more reliable decision making than one based on feelings of familiarity. The accumulation of evidence in the sequential analysis model could be derived from how effective a segment is in predicted future material. The strategy also exploits the information available to dynamic perception.

This sequential analysis approach also admits the occurrence of false positives for patterns that are similar to the target pattern, since it is stochastic and based on partial evidence. Reductions in criterion towards the end of the interval if a target has not been found (as a consequence of an urgency signal in the accumulation model) would explain increases in false alarms towards the end of the test. Importantly, this approach readily accommodates the observation that recognition improves with the length of the target sequence at short durations, as more information about the target allows more evidence to accrue. A consequence of this strategy is that it should be easier to recognise more predictable dynamic events.

## Methods

### Ethics

All experiments were carried out in accordance with relevant guidelines and regulations. All experimental protocols were approved by the UCL Ethics Committee (approval no. CPB/2010/003). All observers were over 18 and provided informed consent.

### Video recording

A continuous 45-min recording was acquired from a log hearth fire using a Sony HXR-NX5E digital camcorder recording at 50 Hz with a shutter speed of 1/150 (the shutter was open for 6.67 ms). The scene was lit by a mixture of natural and artificial light and no CCD gain was applied. Video was saved directly to the compressed AVCHD format at an initial resolution of 1024 $\times$ 768. Before presentation, stimuli were cropped to 564 $\times$ 641 pixels, removing the background and most of the fireplace. Individual frames were decompressed and saved as bitmaps.

### Observers

We recruited observers from an email list operated by UCL Experimental Psychology. Most observers were degree or master’s degree students. To enter the test phase, participants were required to reach a level of accuracy of at least 75% correct during an easy training phase consisting of 20 trials. Only two subjects failed this criterion across all experiments.

### Equipment

The experiments took place in a darkened room. Stimuli were displayed with a frame rate of 85 Hz on a Mitsubishi DiamondPlus 230 SB CRT monitor with a resolution of 1280 $\times$ 1024. The monitor was calibrated using a Cambridge Research Systems ColorCal or ColorCal MKII. The observer’s head was kept still using a chin rest placed 57 cm from the screen. The active video area subtended a visual angle of 14$^\circ$. Observers’ eye movements were not restricted and they could scan the video if they wished and time allowed. Experiments were programmed in Matlab using the Psychtoolbox psychophysics library^34^. Since our video data was recorded at 50 Hz and the monitor refreshed at 85 Hz, we calculated the optimal video frame to display and used Psychtoolbox’s Screen(‘Flip’) function to schedule frame updates.

### Procedure

*Experiment 1* Ten participants were recruited from the UCL Experimental Psychology subject pool. The sample and two tests required for each trial were selected from a randomly-chosen 20-s window of the video dataset, in order to ensure no obvious differences in log position or overall form between the two tests. We selected a 300 ms test sequence randomly from this 2-s window, then extracted a 200 ms target sequence from within the selected test sequence, ensuring that first or final frames did not co-occur. The foil for that trial was another, non-overlapping, 300 ms sequence drawn from the same 20-s region.

Observers viewed a 200 ms test clip that had either been left unaltered, colour reversed in hue by inverting the H channel in HSV space, spatially inverted by rotation, or played backwards. The test was followed by two unprocessed 300 ms clips, one of which contained the target (Fig. 1A). The test intervals were separated by an interstimulus interval of 1 s. Observers were asked to identify which clip contained the target. The test clips were slightly longer (300 ms) than the embedded target (200 ms) to avoid observers basing decisions on iconic memory for the first or last frames. The four conditions were blocked. There were 128 trials per condition.

*Experiment 2* Twelve participants were recruited from the UCL Experimental Psychology subject pool. Experiment 2 followed the same 2IFC procedure as Experiment 1 with the exception that in this case, we varied the number of target frames from 1 to 12 (1, 3, 6, or 12 frames), and embedded them randomly in test sequences of 15, 20, or 40 frames so that first or final frames did not co-occur. Sequences were displayed as recorded. The interstimulus interval was 1 s. The test sequence length varied across blocks and target length varied within blocks. There were 40 trials per condition.

*Experiment 3* Thirteen participants were recruited from the UCL Experimental Psychology subject pool. The experimental set-up was as described for Experiment 1. We used a binary choice task in which participants reported whether they thought a video sequence was played forwards or backwards by a button press. The sample clip was displayed for 2 s. Each sample was drawn from a randomly-selected 20 s section of the original video sequence. The sequence was down-sampled to display rates of 25, 16.7, 12.5, 10, 8.3, 7.1, 6.3, 5.6 and 5 Hz and frames were scheduled at the optimal times using Psychtoolbox. The stimulus duration remained constant, but frames were only updated to the new time-matched stimulus after the appropriate delay. The sequences were displayed in 4 spatial orientations, (0, 90, 180, or 270 degrees). Orientation conditions were blocked and display rate conditions were randomly interleaved. There were 25 trials per condition.

*Experiment 4* Twelve participants were recruited from the UCL Experimental Psychology subject pool. The design of the experiment followed that of Experiment 2. However, the clips were of a longer duration. The target clip could be either 200 ms, 500 ms, or 1000 ms in length. The target clips were embedded in test clips that were 1.2, 1.4, 1.6, 1.8 or 2 times longer than the target. The target could appear at any point in the test sequence with the constraint that it did not start at the beginning (share a first frame) or finish at the end (share a final frame).

*Experiment 5* Eleven participants were recruited from the UCL Experimental Psychology subject pool. We used a Yes/No detection task. Each sample/test pair was drawn randomly from a 200 s sample of the original video sequence. We presented a sample clip followed by a longer test clip. Observers reported whether they thought the target sample was embedded in the test sequence or not. The target was 1 s in duration and could appear at range of time points from the beginning to the end of the sequence. It could be preceded by 0–2 s of video and followed by 0–2 s of video. Thus, the trial could last as long as 5 s. The foils were matched in length.

*Experiment 6* Eight participants were recruited from the UCL Experimental Psychology subject pool. The stimuli were drawn from a 200 s segment of the initial recording. The design of this experiment followed Experiment 5 in using a Yes/No detection task; however, in this case, the 1 s target could appear with a range of delays relative to the onset of the test sequence which was presented for 3 s. Participants indicated whether the target was present or not using a button press.

## Supplementary Information

Supplementary Video 1.

Supplementary Video 2.

Supplementary Video 3.

Supplementary Video 4.

Supplementary Video 5.

Supplementary Video 6.

Supplementary Video 7.

Supplementary Video 8.

## References

[1] Riesenhuber M, Poggio T (1999). Hierarchical models of object recognition in cortex. Nat. Neurosci..

[2] Krizhevsky A, Sutskever I, Hinton GE (2012). Imagenet classification with deep convolutional neural networks. Adv. Neural Inf. Process. Syst..

[3] Marcus, G. Deep learning: A critical appraisal. arXiv preprint arXiv:1801.00631 (2018).

[4] Yuille AL, Liu C (2020). Deep nets: What have they ever done for vision?. Int. J. Comput. Vis..

[5] Serre T (2019). Deep learning: The good, the bad, and the ugly. Annu. Rev. Vis. Sci..

[6] Rousselet G, Joubert O, Fabre-Thorpe M (2005). How long to get to the “gist” of real-world natural scenes?. Vis. Cognit..

[7] Brady TF, Konkle T, Alvarez GA, Oliva A (2008). Visual long-term memory has a massive storage capacity for object details. Proc. Natl. Acad. Sci..

[8] Knight B, Johnston A (1997). The role of movement in face recognition. Vis. Cognit..

[9] Hill H, Johnston A (2001). Categorizing sex and identity from the biological motion of faces. Curr. Biol..

[10] Yovel G, O’Toole AJ (2016). Recognizing people in motion. Trends Cogn. Sci..

[11] Marr D, Nishihara HK (1978). Representation and recognition of the spatial organization of three-dimensional shapes. Proc. R. Soc. Lond. Ser. B. Biol. Sci..

[12] Blanz, V. & Vetter, T. A morphable model for the synthesis of 3d faces. In *Proceedings of the 26th Annual Conference on Computer Graphics and Interactive Techniques*, 187–194 (1999).

[13] Cowe, G. A. *Example-based computer-generated facial mimicry*. Ph.D. thesis, University College London (University of London) (2003).

[14] Chang L, Tsao DY (2017). The code for facial identity in the primate brain. Cell.

[15] Johnston A, Curio C, Bülthoff HH, Giese MA (2011). Is dynamic face perception primary?. Dynamic Faces: Insights from Experiments and Computation (MIT Press.

[16] Kawabe T, Maruya K, Fleming RW, Nishida S (2015). Seeing liquids from visual motion. Vis. Res..

[17] Kawabe T, Maruya K, Nishida S (2015). Perceptual transparency from image deformation. Proc. Natl. Acad. Sci..

[18] Bi W, Jin P, Nienborg H, Xiao B (2018). Estimating mechanical properties of cloth from videos using dense motion trajectories: Human psychophysics and machine learning. J. Vis..

[19] Bi W, Jin P, Nienborg H, Xiao B (2019). Manipulating patterns of dynamic deformation elicits the impression of cloth with varying stiffness. J. Vis..

[20] McDermott JH, Simoncelli EP (2011). Sound texture perception via statistics of the auditory periphery: Evidence from sound synthesis. Neuron.

[21] McDermott JH, Schemitsch M, Simoncelli EP (2013). Summary statistics in auditory perception. Nat. Neurosci..

[22] Doretto G, Chiuso A, Wu YN, Soatto S (2003). Dynamic textures. Int. J. Comput. Vis..

[23] Sperling G (1960). The information available in brief visual presentations. Psychol. Monogr. Gen. Appl..

[24] Breitmeyer BG, Ogmen H (2000). Recent models and findings in visual backward masking: A comparison, review, and update. Percept. Psychophys..

[25] Smithson H, Mollon J (2006). Do masks terminate the icon?. Q. J. Exp. Psychol..

[26] Yu C, Mei Z, Zhang X (2013). A real-time video fire flame and smoke detection algorithm. Proc. Eng..

[27] Zhang B (2020). Deepfirenet: A real-time video fire detection method based on multi-feature fusion. Math. Biosci. Eng. MBE.

[28] Treisman AM, Gelade G (1980). A feature-integration theory of attention. Cognit. Psychol..

[29] Wolfe JM, Horowitz TS (2004). What attributes guide the deployment of visual attention and how do they do it?. Nat. Rev. Neurosci..

[30] Gold, J. I. & Shadlen, M. N. The neural basis of decision making. *Annu. Rev. Neurosci.* **30**, 535–574 (2007). 10.1146/annurev.neuro.29.051605.113038 17600525

[31] Ratcliff R, Huang-Pollock C, McKoon G (2018). Modeling individual differences in the go/no-go task with a diffusion model. Decision.

[32] Ratcliff R, Smith PL, Brown SD, McKoon G (2016). Diffusion decision model: Current issues and history. Trends Cognit. Sci..

[33] Granger, C. W. Investigating causal relations by econometric models and cross-spectral methods. *Econ. J. Econ. Soc.* **37**(3), 424–438 (1969).

[34] Kleiner, M., Brainard, D. & Pelli, D. What’s new in psychtoolbox-3? perception ecvp abstract supplement. *PLOS One* **36**(14), 1–16 (2007).

